# A Retrospective Observational Study of Ephedrine Use in Hip Arthroplasty: Routine Practice at a Secondary Care Hospital in Romania

**DOI:** 10.3390/clinpract15090166

**Published:** 2025-09-15

**Authors:** Erika Bimbo-Szuhai, Mihai Octavian Botea, Harrie Toms John, Adela Bostan Danciu, Pirvan Titus Razvan, Mihaela Gabriela Bontea, Mihai Pavel, Caius Salajan, Maria Viviana Rusu, Adrian Gheorghe Osiceanu, Iulia Codruta Macovei

**Affiliations:** 1 Department of Morphological Disciplines, Faculty of Medicine and Pharmacy, University of Oradea, 1st December Square 10, 410073 Oradea, Romania; pirvantitusrazvan@uoradea.ro (P.T.R.); bonteagabrielamihaela@uoradea.ro (M.G.B.); or osiceanuadrian@uoradea.ro (A.G.O.); 2 Pelican Hospital, Corneliu Coposu Street 2, 410450 Oradea, Romania; mbotea@uoradea.ro (M.O.B.); danciubostanadela@student.uoradea.ro (A.B.D.); cmacovei@uoradea.ro (I.C.M.); 3 Department of Surgical Disciplines, Faculty of Medicine and Pharmacy, University of Oradea, 1st December Square 10, 410073 Oradea, Romania; 4 Faculty of Medicine and Pharmacy, University of Oradea, 1st December Square 10, 410073 Oradea, Romania; salajanharai.caiushoratiuaaron@student.uoradea.ro (C.S.); rusu.marianaviviana@student.uoradea.ro (M.V.R.); 5 Doctoral School of Biomedical Sciences, University of Oradea, 410087 Oradea, Romania; pavel.mihai1@student.uoradea.ro

**Keywords:** anesthesia, total hip arthroplasty, hypotension, spinal

## Abstract

**Background and Objectives:** The primary goal of the study is to analyze factors associated with spinal anesthesia-induced hypotension (SAIH), with a focus on ephedrine requirements in relation to patient characteristics and the type of intrathecal opioid used, reflecting real-world clinical practice in a Romanian secondary care hospital. Bolus ephedrine is often required during spinal anesthesia to maintain hemodynamic stability. We conducted a retrospective observational study of patients undergoing total hip arthroplasty. We analyzed the hemodynamic effects of spinal anesthesia to optimize management of spinal anesthesia-induced hypotension (SAIH). **Materials and Methods:** A total of 329 patients were included in the study, out of which 113 patients were without high blood pressure (60 cases needed Ephedrine) and 216 patients with high blood pressure were drug controlled (106 cases needed Ephedrine). Each group of patients was divided into two groups based on the type of spinal anesthesia: bupivacaine with morphine (Group M) and bupivacaine with fentanyl (Group F). The study explored perioperative factors associated with spinal anesthesia-induced hypotension and the ephedrine dose required to maintain hemodynamic stability. **Results:** We found that ephedrine dosage correlated with hypertension in 19% of cases and with patient age in 44.1% of cases. The type of anesthetic mixture did not significantly affect the need for intraoperative ephedrine administration. **Conclusions:** Ephedrine remains essential for ensuring hemodynamic stability and optimizing perioperative outcomes.

## 1. Introduction

Hip arthroplasty is a common orthopedic procedure that requires careful intraoperative hemodynamic management to ensure patient safety and optimal outcomes. Spinal anesthesia is often used because of its efficacy in pain control and favorable safety profile. However, it is associated with a well-documented risk of hypotension due to sympathetic blockade, often requiring vasopressors such as ephedrine to maintain adequate perfusion [1].

Ephedrine, a mixed α- and β-adrenergic agonist, is widely used to treat intraoperative hypotension due to its dual ability to increase cardiac output and systemic vascular resistance [2]. Ephedrine’s effectiveness may vary depending on the spinal anesthetic used and individual patient factors, such as hypertension. Hypertension-related autonomic dysfunction and vascular remodeling in hip arthroplasty patients may blunt the response to vasopressors [3]. Understanding this relationship is crucial for improving perioperative management strategies.

Specific drug combinations, such as bupivacaine with opioid adjuvants like fentanyl or morphine, influence the depth and duration of sympathetic blockade, which in turn affects hemodynamic responses [4]. While these combinations can improve pain relief, they may also increase the risk of hypotension. Hypotension from spinal anesthesia is primarily caused by sympathetic blockade, and older patients are especially vulnerable due to limited cardiovascular reserve and diminished baroreceptor function [5]. Intraoperative hypotension is not benign; it is associated with myocardial ischemia, renal injury, and cognitive impairment, particularly in elderly or frail patients [6]. Bone Cement Implantation Syndrome (BCIS) may be an under-recognized complication with diverse and potentially severe clinical manifestations [7].

To address these risks, vasopressors like ephedrine, phenylephrine, or norepinephrine are commonly administered. Ephedrine’s dual action—both receptor stimulation and indirect catecholamine release—makes it a versatile agent [8,9]. However, managing spinal anesthesia-induced hypotension becomes more complex in patients with chronic hypertension. Long-standing high blood pressure leads to left ventricular hypertrophy, decreased adrenergic receptor sensitivity, and increased vascular stiffness [10,11]. The use of antihypertensive drugs, including β-blockers and ACE inhibitors, can further complicate intraoperative hemodynamic control [12].

Patients with hypertension may experience more profound hypotension under spinal anesthesia due to impaired compensatory mechanisms [11]. Variations in local anesthetics (e.g., bupivacaine, ropivacaine, lidocaine) and opioid adjuvants (e.g., fentanyl, morphine) also affect vasopressor needs and blood pressure control [13,14,15].

Personalized perioperative care that incorporates individual patient characteristics can enhance anesthetic protocols and vasopressor strategies, improving clinical outcomes in hip arthroplasty.

This study aims to contribute to personalized anesthesia care by identifying patient-related factors—particularly age and hypertension—that influence intraoperative hemodynamic stability and the responsiveness to ephedrine. We hypothesized that these factors would significantly impact the dose of ephedrine required during spinal anesthesia for total hip arthroplasty.

## 2. Materials and Methods

### 2.1. Study Design

We conducted a retrospective, single-center observational study involving 329 patients who underwent orthopedic hip arthroplasty under spinal anesthesia (SA) between January and December 2022. All patients provided written informed consent, in accordance with hospital policies. The study was approved by the Ethics Committee of Pelican Hospital Oradea (approval number 2591/15 December 2021) and conducted in alignment with the Declaration of Helsinki.

### 2.2. Data Collection

Demographic and clinical data were collected retrospectively from electronic medical records, including age, sex, ephedrine dosage, and presence of cardiovascular comorbidities. Anesthetic charts and perioperative hemodynamic data were reviewed. Ephedrine dosage was recorded at the time of administration by the attending anesthesiologist. Hypertension status was confirmed by preoperative cardiology assessment. All evaluations were conducted using standardized hospital protocols and calibrated monitoring equipment.

### 2.3. Variables

The primary outcome variable was the dose of ephedrine (mg) administered intraoperatively to treat spinal anesthesia-induced hypotension. Independent variables included age (years), hypertension status (defined as systolic BP ≥ 140 mmHg and/or diastolic BP ≥ 90 mmHg or ongoing antihypertensive therapy), and type of spinal anesthesia (bupivacaine with morphine vs. bupivacaine with fentanyl) (BP—blood pressure). Categorical variables were coded as binary for statistical analysis. No continuous variables were categorized during data collection; grouping was only applied for descriptive purposes during reporting.

### 2.4. Inclusion Criteria

Eligible patients were aged 18 years or older, had an ASA Physical Status I or II, and were scheduled for hip arthroplasty under spinal anesthesia. Additional inclusion criteria included left ventricular ejection fraction (EF) > 45% and absence of severe aortic stenosis. All patients underwent preoperative evaluation in the outpatient anesthesiology department.

### 2.5. Exclusion Criteria

Exclusion criteria included the following: age under 18, contraindications to spinal anesthesia (patient refusal, infection at the injection site, severe coagulopathy, elevated intracranial pressure, and true allergy to the drugs), history of revision surgery, presence of implanted devices for instability, hemodynamic instability, and refusal to participate.

### 2.6. Anesthetic Protocol and Monitoring

Pre-anesthetic evaluation was conducted two weeks prior to surgery, and patients were hospitalized one day before the procedure. Hypertensive patients were identified through cardiology consultation, meeting the criteria of SBP (Systolic Blood Pressure) ≥ 140 mmHg and/or DBP (Diastolic Blood Pressure) ≥ 90 mmHg, and were managed as per the American Heart Association (AHA) guidelines. Only β-blockers were continued on the day of surgery. Alprazolam 0.5 mg was administered the night before for anxiolysis.

In the operating room, an 18-G IV cannula was inserted, and Ringer’s lactate (15 mL/kg) was administered. Premedication included 2 mg midazolam and 1 g tranexamic acid. Following skin disinfection and local infiltration with 1% lidocaine, spinal anesthesia was administered via the paramedian approach at L3–L4 using a 26G Sprotte needle (PAJUNK GmbH, Geisingen, Germany). Patients received hyperbaric bupivacaine 0.5% (15–17.5 mg) combined with either 25 mcg fentanyl or 150 mcg morphine, delivered within 15 s.

Oxygen was administered at 5 L/min via face mask. Sensory blockade was evaluated five minutes post-injection using ice or pinprick stimuli. Patients were then placed in the dorsal decubitus position, and intraoperative monitoring included ECG, noninvasive blood pressure (every 5 min), pulse oximetry (SpO_2_), and respiratory rate. All patients underwent anterior approach total hip arthroplasty, performed by the same surgical team, with blood loss under 200 mL.

Hemodynamic rescue protocol included the following:

1. Severe hypotension was defined as a systolic blood pressure (SBP) < 90 mmHg or a decrease of more than 30% from baseline within the first 45 min after spinal anesthesia. When these criteria were met, patients were treated with 5 mg intravenous ephedrine boluses administered every 5 min, in combination with additional Ringer’s lactate infusion, until SBP reached ≥110 mmHg. This protocol was consistently applied to all patients during the intraoperative period and documented by the attending anesthesiologist in the anesthesia record.
2. Bradycardia (HR ≤ 50 bpm): 0.5 mg atropine IV, repeated as necessary every minute, up to a maximum 2 mg total dose.

### 2.7. Statistical Analysis

**Figure 1:**
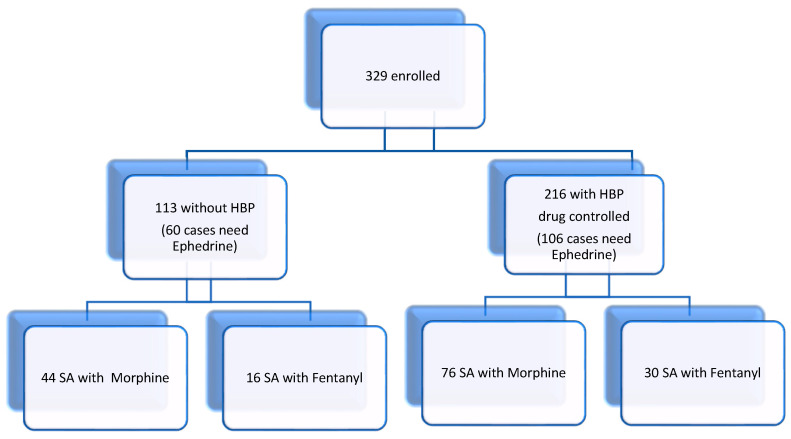
Flow chart (HBP—high blood pressure).

Statistical data were processed with IBM SPSS version 23. The Chi-Square test was applied to assess the significance of the relationship between the categorical variables (age and hypertension). We employed Eta squared measures to determine the extent of the total variance in the dependent variable (Ephedrine) that can be attributed to the different group membership (bupivacaine with morphine—group M, and bupivacaine with fentanyl—group F). Missing data were minimal (<5%) and excluded from analysis using pairwise deletion. No imputation methods were applied, due to the limited scope of missingness. Assumptions for regression models (linearity, multicollinearity, and homoscedasticity) were tested prior to interpretation. No interaction terms were included due to limited subgroup sample sizes, but future work should explore these relationships in larger cohorts. The study cohort selection process is summarized in Figure 1.

### 2.8. Sample Size Justification

The study included all consecutive patients undergoing hip arthroplasty under spinal anesthesia during the 12-month period (*n* = 329). A formal sample size calculation was not performed a priori. Post hoc analysis indicates this sample provides adequate power (>80%) to detect moderate effect sizes (Cohen’s d = 0.3–0.5) with a significance level of 0.05.

### 2.9. Bias Control

As a retrospective study, selection bias was minimized by including all eligible patients within the specified time frame who met the inclusion criteria. Information bias was reduced through standardized data abstraction by trained personnel, using predefined variable definitions. However, unmeasured confounding remains possible due to lack of data on intraoperative blood pressure variability and other comorbidities.

## 3. Results

### 3.1. Correlation Between Ephedrine Dose and Blood Pressure

**Figure 2:**
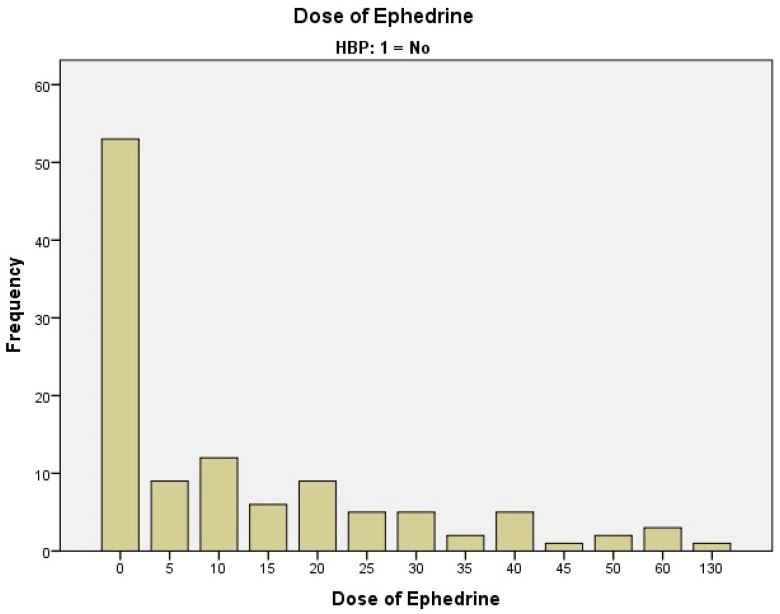
Ephedrine dose used on patients without HBP in correlation with the number of patients (HBP—high blood pressure; frequency represents number of cases).

**Figure 3:**
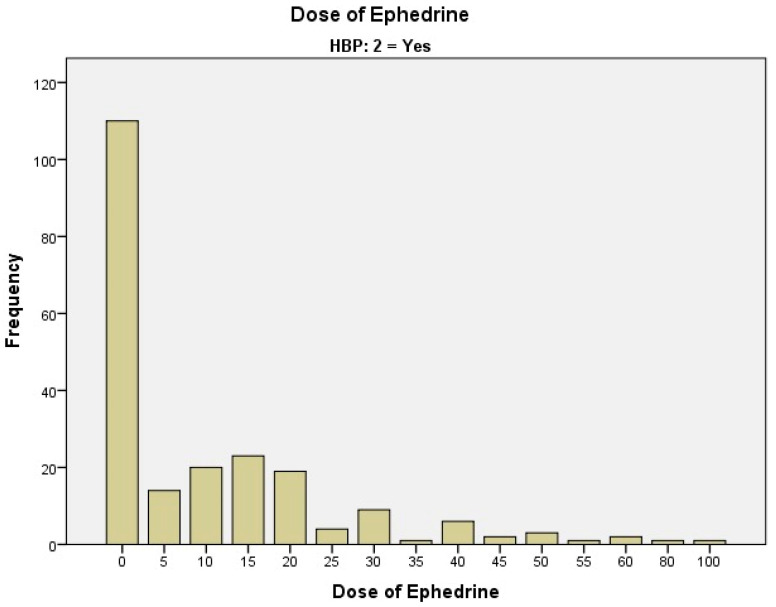
Ephedrine dose used on patients with HBP in correlation with the number of patients (HBP—high blood pressure; frequency represents the number of cases).

We studied 329 patients who were set to undergo hip arthroplasty using spinal anesthesia and assessed the necessary dose of ephedrine for maintaining hemodynamic stability (Table 1, Figure 2 and Figure 3). The most frequently used dose of Ephedrine for intraoperative hemodynamic balancing was 10 mg (12 cases of patients without HBP and 20 cases with HBP), respectively, and 15 mg of Ephedrine (6 cases without HBP and 23 cases with HBP).

This table illustrates the percentage of ephedrine doses in relation to the total number of patients enrolled in this study, with or without HBP. Overall, as for the trend in ephedrine use, patients with HBP generally received ephedrine more frequently across most dosage levels compared to patients without HBP. At nearly every dose level, the percentage of HBP patients receiving ephedrine is equal to or higher than the percentage in the non-HBP group. HBP patients tend to receive ephedrine more frequently than those without HBP. Ephedrine use decreases as the dose of ephedrine increases.

As shown in the table above (Table 1), the groups of hypertensive patients versus non-hypertensive patients are distributed as in the flowchart and figures below (Figure 2 and Figure 3).

The use of ephedrine in these non-hypertensive patients (Figure 2) is predominantly avoided or used in low doses, and the graphics show a conservative approach to ephedrine administration in this group, to avoid adverse effects. High doses are exceptions, and are used only in special cases. The differences between the two groups (Figure 2 and Figure 3) suggest that, in general, high-dose ephedrine is administered less frequently to patients with hypertension, and low doses are used more frequently, especially in the context of cardiovascular risks.

As Table 2 shows, in patients with normal blood pressure, Ephedrine was administered in 60 cases (53.1%), and in hypertensive patients, it was necessary in 106 cases (49.1%). This demonstrates there is no statistically significant difference between the two groups.

A Chi-Square test of independence run for the study group showed that there was no significant association between the two groups with a Pearson Chi-Square test of 0.480^a^, a Fisher’s Test = 0.562, and a Likelihood Ratio = 0.481.

Because we did not find any significant correlations in the Chi-Square analysis, we also carried out an ETA statistical test in the data analysis, and we observed that in 19% of the cases HBP is correlated with the amount of ephedrine dose, which is a value with practical significance. The reason we choose the ETA coefficient (a cross-tab correlation analysis) is based on the variable type. One variable is on a nominal scale, and the other one is an interval (numeric) measure scale, 19%, representing a small-to-medium-sized effect. Table 1 shows that in 53 patients without HBP and 110 patients with HBP, the use of Ephedrine during the intervention was not necessary.

### 3.2. Correlation Between Ephedrine Dose and Age

**Figure 4:**
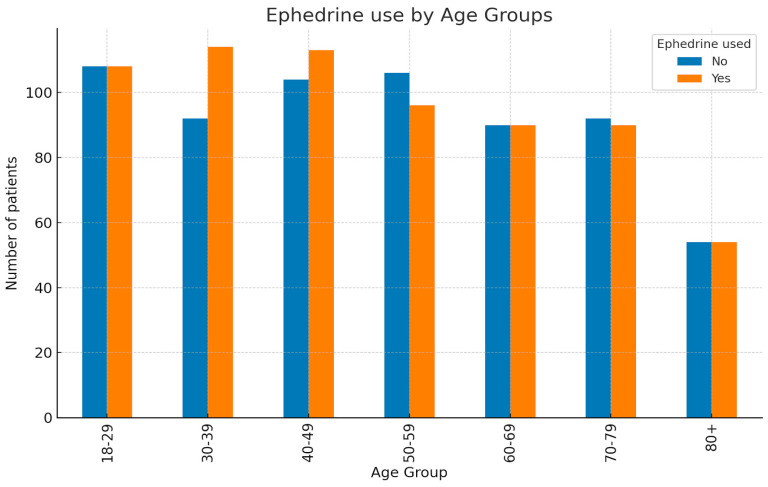
The age of patients correlated with the Ephedrine used.

A bar chart was generated to illustrate the distribution of patients who did or did not receive ephedrine across different age groups as shown in Figure 4.

Overall, the number of patients was similar between the “No ephedrine” use and “Yes ephedrine” use groups. The largest proportions of patients were observed in the younger (18–49 years) and middle-aged (50–59 years) groups, whereas the smallest numbers were seen in the oldest age group (80+ years). The age distribution pattern was comparable between those who received ephedrine and those who did not, indicating that ephedrine use was relatively evenly distributed across age categories.

Following ETA data analysis, we conclude that age correlates with the dose of Ephedrine.

As we observe, in 44.1% of cases, the dose of Ephedrine is correlated with the age of the patient, which is a value with practical significance.

### 3.3. Correlation Between Ephedrine Dose and Type of SA

We studied 329 patients scheduled for orthopedic hip arthroplasty surgery under SA with morphine and bupivacaine versus SA with fentanyl and bupivacaine, and we compared the required intraoperative ephedrine use for hemodynamic patient stabilization (Table 3).

As we can see in Table 3, in the case of SA group F, the administration of Ephedrine was necessary in 46 cases (50.5%), and in the case of SA group M, it was necessary in 120 cases (50.4%), a fact that demonstrates there is no significant difference in percentage.

In the statistical study conducted using the Chi-Square Test, we aimed to determine if there is a statistically significant difference between the dose of Ephedrine and the type of anesthesia. The values obtained demonstrate their independence (Pearson Chi-Square = 0.983, Fisher’s Exact Test = 1, Likelihood Ratio = 0.983, N (sample size) = 329). There is no significant relationship between the two variables.

### 3.4. Multivariate Analysis

Logistic regression indicated that age was significantly associated with higher odds of receiving ephedrine (or: 1.04, 95% CI: 1.01–1.07, *p* = 0.015), while hypertension was not a significant independent predictor (or: 1.08, 95% CI: 0.70–1.66, *p* = 0.73). the type of intrathecal adjuvant (morphine versus fentanyl) was not significantly associated with ephedrine use (or: 1.02, 95% CI: 0.63–1.64, *p* = 0.93).

In linear regression, age remained a significant predictor of ephedrine dose (β = 0.18, *p* = 0.004), explaining approximately 5.4% of the variance. Hypertension and type of anesthesia were not statistically significant predictors.

## 4. Discussion

In our study, we considered patients with hypertension (HBP) a high-risk factor for spinal anesthesia-induced hypotension (SAIH) because we observed that in 19% of the cases, HBP was correlated with the amount of ephedrine dose used, which is a statistical value with practical significance. Our study also examines how the administration of ephedrine prevents hypotension after SA and impacts hemodynamic changes in elderly patients. In 44.1% of our cases, the dose of ephedrine was correlated with age, indicating a value with practical significance.

The variability in ephedrine requirements among patients highlights the need for a more personalized approach to spinal anesthesia management. Patients experience variations in vasopressor response due to their baseline autonomic tone, arterial stiffness from hypertension, and cardiovascular system changes associated with aging [10,12]. Preoperative patient analysis using advanced machine learning algorithms could determine customized vasopressor doses based on blood pressure variability, heart rate dynamics, and pharmacokinetic data, enhancing operative safety and postoperative recovery [14].

Spinal anesthesia is a commonly used technique, as it is accessible, safe, and easy to perform. Before the surgery, each patient was informed regarding the details of the surgical approach and possible intraoperative hemodynamic variations induced by SA. For optimal surgical anesthesia, a local anesthetic agent can induce adequate relaxation of the lower limb muscles. By combining opioids with bupivacaine, a decrease in the local anesthetic dose can be achieved with similar efficiency and improved hemodynamic stability [16,17,18].

Hypotension induced by spinal anesthesia frequently occurs in elderly patients. Many studies have been performed to decrease the incidence of SAIH, but none have proved sufficiently compelling [19]. Proper sedation is now a key aspect of spinal anesthesia, vital for ensuring patient comfort and improving compliance with regional anesthesia techniques [19].

Among spinal anesthesia side effects, cardiovascular complications are the most prominent, with hypotension being considered the most common, observed in up to 33% of patients [20]. SA influences the sympathetic chain, causing reduced vasomotor tone, which leads to reduced preload (from vasodilation and decreased venous return), diminished afterload (due to lower systemic vascular resistance) [21,22], and, ultimately, reduced cardiac output, particularly in older adults [22].

The leading cause of hypotension is the total dose of anesthetic administered. However, multiple other factors, such as the anesthetic type, its volume, accompanying adjuvant agents, and preoperative or intraoperative variables, also contribute to SA’s hemodynamic effects [22,23].

Our study compared SA group F with SA group M and the number of cases that needed ephedrine for hemodynamic stability. There was no significant difference in percentage between the two groups. No major differences emerged from choosing bupivacaine–morphine or bupivacaine–fentanyl in this study group, regarding ephedrine demand. Additional investigations should examine customized vasopressor dose frequencies to match each patient’s preoperative blood pressure dynamics, for better clinical results.

For a long time, ephedrine has been considered the standard vasoconstrictor for hypotension induced by SA [24,25]. Ephedrine, a widely used adrenergic stimulant with both direct and indirect actions, has traditionally been favored for addressing the undesired hemodynamic changes caused by spinal anesthesia [25]. Administering ephedrine using repeated intermittent IV boluses, however, is reactive rather than preventive, leading to fluctuating drug levels between peaks and troughs [26]. Ephedrine is a sympathomimetic drug commonly used in perioperative settings, particularly during anesthesia, to manage hypotension that can occur after induction or during surgery [27].

Ephedrine, a mixed-action adrenergic agonist (targeting both alpha and beta receptors), increases cardiac output while mitigating heart rate reduction. It can achieve this by causing vasoconstriction that surpasses arteriolar constriction, improving venous return (preload), and boosting cardiac output, blood pressure, and heart rate [27]. Various studies indicate that 13% of non-obstetrical patients develop bradycardia during SA [28], and, if corrective measures are taken, there are no significant consequences [29].

Hypotension following spinal anesthesia is primarily due to sympathetic blockade, leading to vasodilation and a subsequent reduction in systemic vascular resistance (SVR). Additionally, venous pooling in the lower extremities and decreased cardiac preload contribute to reduced cardiac output. These effects necessitate prompt and effective management to maintain adequate perfusion, especially of vital organs [30].

Some studies describe the fact that in SA, arterial vasodilatation reaches a maximum after about 7 min [30,31]. Age-related changes, such as alterations in systolic function and diastolic relaxation in the elderly, can aggravate the decrease in cardiac output [32,33,34,35].

Hypotension resulting from spinal anesthesia, primarily due to reduced stroke volume, can be managed with crystalloid or colloid solutions. Vasopressor agents, such as ephedrine or phenylephrine, increase stroke volume and preload by increasing systemic vascular resistance without direct heart stimulation [26,27]. Thus, vasopressors combined with crystalloid solutions effectively treat post-subarachnoid block hypotension in elderly patients.

Our research focused on the role of ephedrine in preventing hypotension following spinal anesthesia and its effects on hemodynamic changes in elderly patients. In 44.1% of the instances, we found a meaningful connection between the dosage of ephedrine and age. Various studies have highlighted the critical role of avoiding even short episodes of hypotension in elderly patients to reduce the risks of complications and mortality [36,37]. It is a well-known fact that the incidence of hypotension induced by SA increases with age (about 36% of younger patients, rising to 75% of patients over the age of 50) [38]. Even with low doses of bupivacaine, hypotension induced by SA remains high in older patients [33]. Mon et al. have shown that ephedrine administration was associated with reasonable systolic blood pressure control [39].

Spinal anesthesia, a commonly used regional anesthesia technique, is widely employed for surgeries involving the lower abdomen, pelvis, and lower extremities. In orthopedic surgery, to minimize the hemodynamic consequences of SA, the patient’s position can be lateralized to the side to be operated on to obtain a predominantly unilateral sympathetic block [40,41]. This can be helpful in elderly patients who are at a higher risk of SAIH.

As a single-center study conducted in a Romanian tertiary care hospital, findings may not be generalizable to all populations, particularly settings with different anesthetic protocols or availability of vasopressors. However, the physiological mechanisms underlying spinal anesthesia-induced hypotension and ephedrine response are broadly applicable across surgical contexts and patient populations.

## 5. Limitations

This study is limited by its retrospective, single-center design. Additionally, phenylephrine was not available, limiting comparisons with other vasopressors. Standardized opioid doses were used, which may not reflect real-world variability. Despite these limitations, the findings provide relevant insights into patient-specific vasopressor management during spinal anesthesia.

## 6. Conclusions

Ephedrine remains a cornerstone in managing hypotension following spinal anesthesia. Its dual mechanism of action and proven efficacy make it a valuable tool in the anesthesiologist’s arsenal. However, individualized patient assessment and careful monitoring are essential to optimize its use and minimize potential side effects. Future research may further refine the role of Ephedrine and other vasopressors in this context, ensuring safer and more effective perioperative care.

This study emphasizes how individualized blood pressure management practices provide essential care for patients during spinal anesthesia procedures of hip arthroplasty. The efficacy of ephedrine administration for managing intraoperative hypotension depends on individual characteristics such as age, blood pressure status, and the natural state of autonomic management in patients. The findings demonstrate the crucial requirement for developing individual spinal anesthesia procedures that would integrate knowledge of patient comorbidities and drug reaction patterns.

Because this was a single-center study conducted in a Romanian tertiary care hospital, the findings may have limited generalizability, particularly in healthcare systems with different anesthetic protocols or access to vasopressors. Therefore, the proposed protocol is best suited for contexts where medication availability and cost represent significant constraints.

Future investigations need to study how pharmacogenomics and real-time hemodynamic monitoring, and AI-based predictive modeling can optimize perioperative vasopressor management. Anesthesiologists can customize their anesthesia protocols using precision medicine approaches to reduce intraoperative complications while ensuring patient safety throughout the recovery process. These advancements follow the patient-centered strategy of personalized medicine that uses individual genetic and physiological profiles to create anesthetic plans.

## Figures and Tables

**Table 1:** Crosstabulation of HBP (HBP—high blood pressure) and the dosage of ephedrine (mg) used.

		Dose of Ephedrine (mg)															
		0	5	10	15	20	25	30	35	40	45	50	55	60	80	100	130
Without HBP	Patient number	53	9	12	6	9	5	5	2	5	1	2	0	3	0	0	1
	Ephedrine (%)	32.5	39.1	37.5	20.7	32.1	55.6	35.7	66.7	45.5	33.3	40	0	60	0	0	100
With HBP	Patient number	110	14	20	23	19	4	9	1	6	2	3	1	2	1	1	0
	Ephedrine (%)	67.5	60.9	62.5	79.3	67.9	44.4	64.3	33.3	54.5	66.7	60	100	40	100	100	0

**Table 2:** Cross tabulation of patients with or without HBP and their requirement for Ephedrine use (HBP—high blood pressure). * denotes a cross-analysis between the two variables.

BP * Ephedrine Crosstabulation					
			Ephedrine Use		Total
			1 = No	2 = Yes	
BP	1 = No	Count	53	60	113
		% with HBP	46.9%	53.1%	100.0%
		% with Ephedrine	32.5%	36.1%	34.3%
		% of Total	16.1%	18.2%	34.3%
	2 = Yes	Count	110	106	216
		% with HBP	50.9%	49.1%	100.0%
		% with Ephedrine	67.5%	63.9%	65.7%
		% of Total	33.4%	32.2%	65.7%
Total		Count	163	166	329
		% with HBP	49.5%	50.5%	100.0%
		% with Ephedrine	100.0%	100.0%	100.0%
		% of Total	49.5%	50.5%	100.0%

**Table 3:** Cross tabulation of SA group F and SA group M and the necessity of ephedrine use. * denotes a cross-analysis between the two variables.

Anesthesia * Ephedrine Cross Tabulation					
			Ephedrine		Total
			1 = No	2 = Yes	
Anesthesia	1 = Fentanyl	Count	45	46	91
		% with SA group F	49.5%	50.5%	100.0%
		% with Ephedrine	27.6%	27.7%	27.7%
		% of Total	13.7%	14.0%	27.7%
	2 = Morphine	Count	118	120	238
		% with SA group M	49.6%	50.4%	100.0%
		% within Ephedrine	72.4%	72.3%	72.3%
		% of Total	35.9%	36.5%	72.3%
Total		Count	163	166	329
		% with SA group F and SA group M	49.5%	50.5%	100.0%
		% with Ephedrine	100.0%	100.0%	100.0%
		% of Total	49.5%	50.5%	100.0%

## Data Availability

Raw data will be made available by the first author without undue reservation.
